# Evaluation of a Vendor-Agnostic Deep Learning Model for Noise Reduction and Image Quality Improvement in Dental CBCT

**DOI:** 10.3390/diagnostics14212410

**Published:** 2024-10-29

**Authors:** Wojciech Kazimierczak, Róża Wajer, Oskar Komisarek, Marta Dyszkiewicz-Konwińska, Adrian Wajer, Natalia Kazimierczak, Joanna Janiszewska-Olszowska, Zbigniew Serafin

**Affiliations:** 1 Department of Radiology and Diagnostic Imaging, Collegium Medicum, Nicolaus Copernicus University in Torun, Jagiellońska 13-15, 85-067 Bydgoszcz, Poland; 2 Department of Radiology and Diagnostic Imaging, University Hospital No. 1 in Bydgoszcz, Marii Skłodowskiej—Curie 9, 85-094 Bydgoszcz, Poland; 3 Kazimierczak Private Medical Practice, Dworcowa 13/u6a, 85-009 Bydgoszcz, Poland; 4 Department of Otolaryngology, Audiology and Phoniatrics, Collegium Medicum, Nicolaus Copernicus University in Torun, Jagiellońska 13-15, 85-067 Bydgoszcz, Poland; 5 Department of Diagnostic Imaging, Poznan University of Medical Sciences, 61-701 Poznań, Poland; 6 Dental Primus, Poznańska 18, 88-100 Inowrocław, Poland; 7 Department of Interdisciplinary Dentistry, Pomeranian Medical University in Szczecin, Al. Powstańców Wlkp. 72, 70-111 Szczecin, Poland; 8 Faculty of Medicine, Bydgoszcz University of Science and Technology, Kaliskiego 7, 85-796 Bydgoszcz, Poland

**Keywords:** cone-beam computed tomography, deep learning model, image quality, noise reduction, dental imaging, oral diagnosis

## Abstract

Background/Objectives: To assess the impact of a vendor-agnostic deep learning model (DLM) on image quality parameters and noise reduction in dental cone-beam computed tomography (CBCT) reconstructions. Methods: This retrospective study was conducted on CBCT scans of 93 patients (41 males and 52 females, mean age 41.2 years, SD 15.8 years) from a single center using the inclusion criteria of standard radiation dose protocol images. Objective and subjective image quality was assessed in three predefined landmarks through contrast-to-noise ratio (CNR) measurements and visual assessment using a 5-point scale by three experienced readers. The inter-reader reliability and repeatability were calculated. Results: Eighty patients (30 males and 50 females; mean age 41.5 years, SD 15.94 years) were included in this study. The CNR in DLM reconstructions was significantly greater than in native reconstructions, and the mean CNR in regions of interest 1-3 (ROI1-3) in DLM images was 11.12 ± 9.29, while in the case of native reconstructions, it was 7.64 ± 4.33 (*p* < 0.001). The noise level in native reconstructions was significantly higher than in the DLM reconstructions, and the mean noise level in ROI1-3 in native images was 45.83 ± 25.89, while in the case of DLM reconstructions, it was 35.61 ± 24.28 (*p* < 0.05). Subjective image quality assessment revealed no statistically significant differences between native and DLM reconstructions. Conclusions: The use of deep learning-based image reconstruction algorithms for CBCT imaging of the oral cavity can improve image quality by enhancing the CNR and lowering the noise.

## 1. Introduction

Cone-beam computed tomography (CBCT) has emerged as a valuable dental imaging tool because of its ability to provide precise three-dimensional reconstruction of the dentomaxillofacial region. CBCT surpasses the limitations of conventional two-dimensional dental imaging, facilitating accurate insight into the multiplanar details of maxillofacial bony structures and adjacent soft tissues. A spatial resolution of less than 100 µm significantly surpasses the imaging capabilities of conventional computed tomography (CT), allowing for precise diagnosis and measurements [1,2,3]. Such precision is desired in implant procedure planning, cephalometry, and endodontics. Although relatively recently introduced (2000s) for broader commercial use, CBCT has already proven its value in a wide range of dental applications, including implant planning, periodontology, temporomandibular joint (TMJ) imaging, orthodontics, and oral and maxillofacial surgery [4,5].

However, the application of CBCT as an imaging modality has limitations. Despite the exceptional image quality achieved in phantom studies, patient studies should be conducted in accordance with ALADIP (As Low as Diagnostically Acceptable being Indication-oriented and Patient-specific) principles [6]. This approach, inter alia, aims to prevent excessive tube setting and thus may lead to a greater number of artifacts and excessive noise. In the case of CBCT, there is a significant variation in image quality, specifically regarding contrast resolution and the level of noise, across various CBCT machines and settings used during acquisition, accompanied by a broad spectrum of radiation doses administered to patients [7]. CBCT artifacts are induced by discrepancies between mathematical models and actual imaging processes [8]. Noise, an unwanted disturbance in a signal, can significantly impair the quality of the images produced by CBCT units. Noise manifests as inconsistent attenuation values in projection images, causing errors in the computed attenuation coefficient and reducing low-contrast resolution, affecting the differentiation of low-density tissues [9,10]. Both artifacts and noise may simulate or obscure pathologies, leading to misdiagnoses and potentially worsening patient outcomes. Additionally, noise is inherently associated with the dose delivered during examinations, demonstrating an inversely proportional relationship [11]. Therefore, it is reasonable to seek noise and artifact reduction, as their application may have an impact on reducing the radiation dose delivered during CBCT and improving its diagnostic accuracy.

To date, several studies have demonstrated the efficacy of deep learning-based image reconstruction algorithms in reducing noise and improving image quality in CBCT scans. For instance, iterative reconstruction (IR) techniques have been shown to significantly enhance image quality in conventional CT and CBCT imaging [12,13,14,15,16,17,18]. Recent advancements in vendor-specific DLRs, such as TrueFidelity™ by GE Healthcare and AiCE by Canon Medical Systems, have further improved diagnostic accuracy and reduced radiation doses [19,20,21]. However, the limitation of these approaches is their vendor-specific nature, which restricts their use to specific scanners. Our study explored the potential of a vendor-agnostic DLM to overcome these limitations. The solution appears to be a vendor-agnostic deep learning model (DLM) that works in the image postprocessing domain and does not require projection data. The term vendor-agnostic refers to the fact that the program is not limited to specific CT or CBCT machine manufacturers and can be applied across different platforms [22]. Previous studies have already proven that vendor-agnostic DLMs can both reduce image noise and provide high diagnostic accuracy comparable to vendor-specific DLRs [23,24,25,26]. Hypothetically, they could also positively affect the quality parameters of dental CBCT images, thereby increasing their diagnostic value for the evaluation of common pathological and dental lesions.

The aim of this study was to assess objective and subjective image quality parameters of standard dental CBCT and DLM-reconstructed images.

## 2. Materials and Methods

### 2.1. Population

The study population consisted of 93 patients (41 males and 52 females aged 15–72 years, SD 15.8; median 41.2). All CBCT scans were acquired at a single private orthodontic center. All patients were referred for CBCT scans by orthodontists and dental surgeons between January and September 2023. The primary indication was suspicion of periapical lesions on the basis of the OPG and single-tooth X-rays. The main study inclusion criterion was images obtained using the standard radiation dose and image quality protocol. Images burdened by motion artifacts were excluded from the study.

### 2.2. Image Acquisition and Postprocessing

All scans were performed using a Hyperion X9 PRO 13 × 10 (MyRay, Imola, Italy). One standard, marked as the “Regular” setting of the apparatus, was used (90 kV, 36 mAs, CTDI/Vol 4.09 mGy, and 13 cm field of view). All images were reconstructed at a slice thickness of 0.3 mm. After scanning, the images were anonymized and exported for further analysis. The deep learning and denoised reconstructions were obtained with the use of ClariCT.AI software (ClariPI, Seoul, Republic of Korea).

### 2.3. Objective Image Quality

To assess the objective image quality, a radiologist with 2 years of craniofacial CT assessment placed square regions of interest (ROIs) at:

1. Periapical region of tooth 15 within the maxillary bone,
2. Periapical region of tooth 33 within the mandible,
3. The spongious bone of the mandible in the mental foramen area,
4. Muscles of the tongue.

The ROIs were carefully placed in homogeneous tissues (spongious bone of periapical regions, mandible, and tongue musculature) to avoid artifacts and lesions (e.g., cysts, enostoses, and endodontic materials). The contrast-to-noise ratio (CNR) was evaluated using ImageJ software v. 1.41 (National Institutes of Health, Bethesda, MD, USA). ROIs were automatically propagated between the native and DLM reconstructions to maximize the objectivity of the results. The CNR calculation formula presented by Koivisto [27] was adopted:

CNR = (S_R1-3,L_ − S_T_)/N

where S_R1-3,L_ is the mean signal at the anatomical landmark or periapical lesion, S_T_ is the mean signal in the background (tongue), and N is the average standard deviation (SD) in the anatomical landmark and background ROI (tongue).

The CNRs of the specified anatomical landmarks were compared to evaluate the effectiveness of the AI denoising tool.

### 2.4. Subjective Image Quality

Subjective image quality was assessed by a radiologist and two dentists (all readers with >5 years of experience in craniofacial CT assessment) who were blinded to patient details and the use of the AI denoising tool. The images were evaluated on a five-point scale (1 = poor, 5 = excellent), considering factors such as noise, sharpness, and visibility of anatomical structures as follows:

Level 5—excellent delineation of structures and excellent image quality;

Level 4—clear delineation of structures and good image quality;

Level 3—anatomical structures still fully assessable in all parts and acceptable image quality;

Level 2—structures identifiable with adequate image quality;

Level 1—anatomical structures not identifiable, images with no diagnostic value.

Image quality assessment was performed in the following predefined anatomical regions: the alveolar recess of the maxillary sinuses, the apical area of tooth 15, and the apical area of tooth 33.

**Figure 1:**
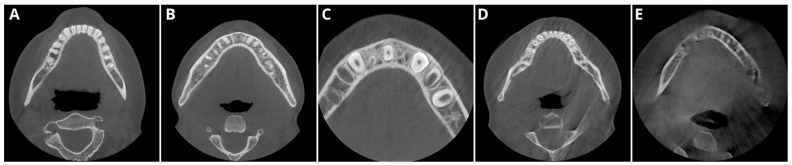
Qualitative image analysis: (**A**)—(5 points) excellent delineation of structures and excellent image quality; (**B**)—(4 points) clear delineation of structures and good image quality; (**C**)—(3 points) anatomical structures still fully assessable in all parts and acceptable image quality; (**D**)—(2 points) structures identifiable in adequate image quality; (**E**)—(1 point) anatomical structures not identifiable, image of no diagnostic value.

To enhance the repeatability and objectivity of the qualitative analyses, an illustration was created to depict representative images evaluated according to the aforementioned scale (Figure 1). In cases of metal artifacts or missing teeth, the opposite side of the dental arch was assessed (e.g., severe artifacts in the apical area of tooth 15–tooth 25 were evaluated).

Agreement between all the readers’ ratings of the subjective image quality of the native and DLM-reconstructed images was assessed.

Subjective image quality analysis was performed on a dedicated console using iRYS Viewer version 6.2 (MyRay, Imola, Italy) software. The window width and center were predefined at 1048 and 4096, respectively.

### 2.5. Error Study

Fifteen randomly selected subjects were re-examined by the same author one month after the initial analysis. The ICC for subjective image quality analyses was calculated to assess the agreement between examinations.

### 2.6. Sample Size Calculation

The post hoc power analysis was conducted to determine adequacy of study sample. A two-tailed paired-sample *t*-test was used since CNR measurements were taken from the same patients under both conditions (native and DLM reconstructions). The effect size (Cohen’s d) for paired samples was assessed with pooled SD of mean CNR values in DLM and native reconstructions. Power analysis was conducted with G*Power software (version 3.1) [28]. The following assumptions were made: α error probability: 0.05, Power (1 − β): 0.80.

### 2.7. Statistical Evaluation

Inter-rater agreement was assessed using Fleiss’ Kappa. Differences between native and DLM reconstructions were analyzed using paired *t*-tests. A power analysis was conducted to determine the appropriate sample size for detecting significant differences in noise levels between the two reconstruction methods. Statistical significance was set at *p* < 0.05 [27]. Statistical analyses were conducted using R software version 4.3.2 [29].

## 3. Results

### 3.1. Population

**Figure 2:**
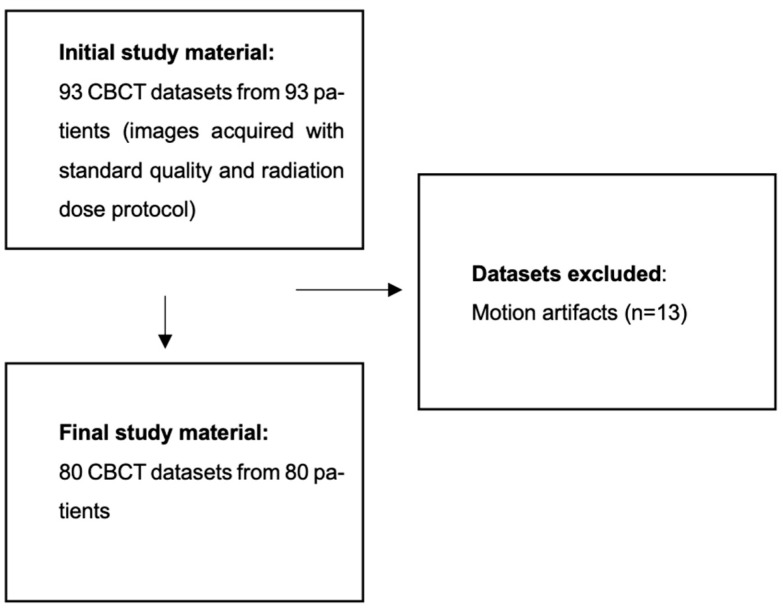
Flow-chart presenting application of eligibility criteria in study material.

The authors screened a total of 93 CBCT scans. Out of these, 13 scans were excluded, as they did not meet the inclusion criteria. Therefore, CBCT scans from 80 patients (30 males and 50 females; mean age 41.45 years, SD 15.94 years) were included in the final analysis (80/93 screened patients). The application of eligibility criteria is presented in Figure 2.

### 3.2. Objective Image Quality

**Figure 3:**
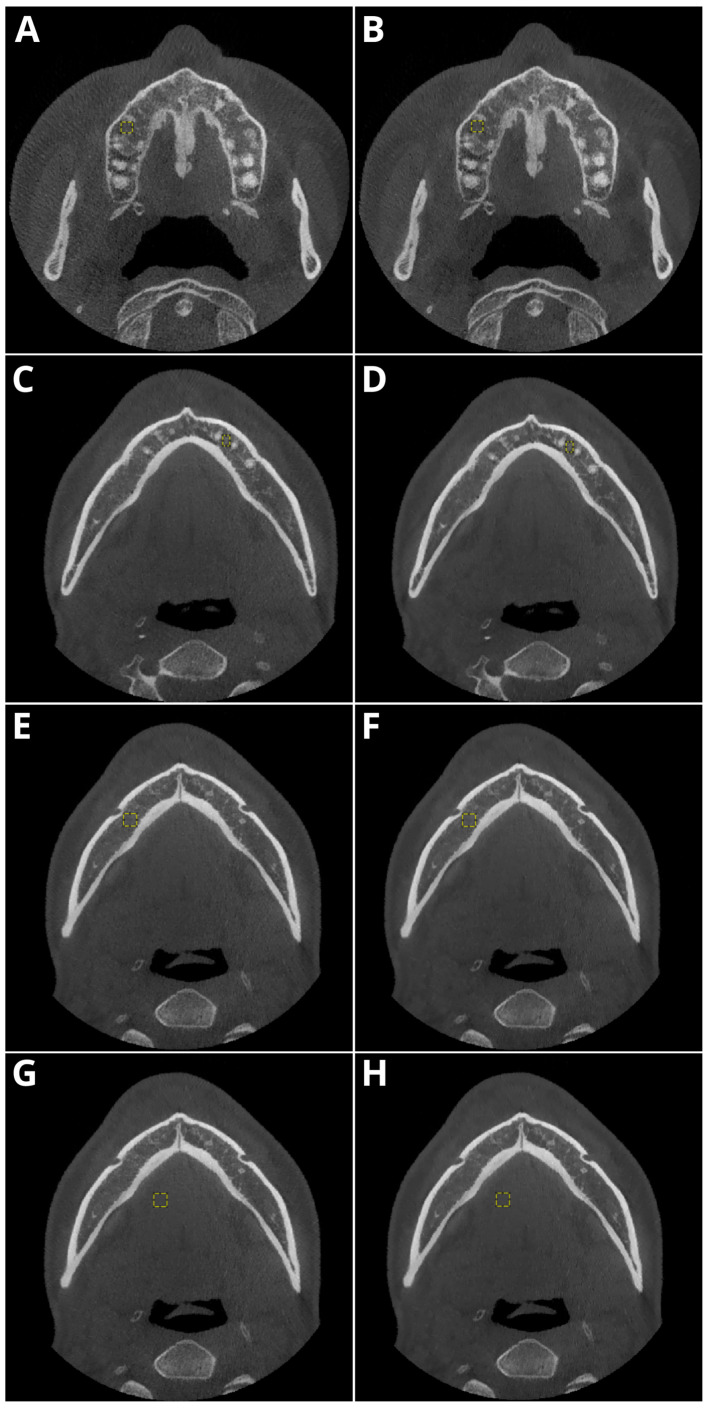
The sample ROI (yellow circle) positions and values in the native (**A**,**C**,**E**,**G**) and DLM (**B**,**D**,**F**,**H**) reconstructions were as follows: (**A**,**B**), tooth 15, mean signal 227.748, 227.267 and SD 179.793, 170,854, respectively; (**C**,**D**), tooth 33, mean signal 418.06, 417.462 and SD 136.493, 129,878, respectively; (**E**,**F**), mental foramen, mean signal 336.191, 330.893 and SD 111.672, 89.153, respectively; and (**G**,**H**), tongue musculature, mean signal 96.336, 95.785 and SD 38.251, 26.848, respectively.

Figure 3 shows the sample ROI position with the corresponding signal and SD values.

**Figure 4:**
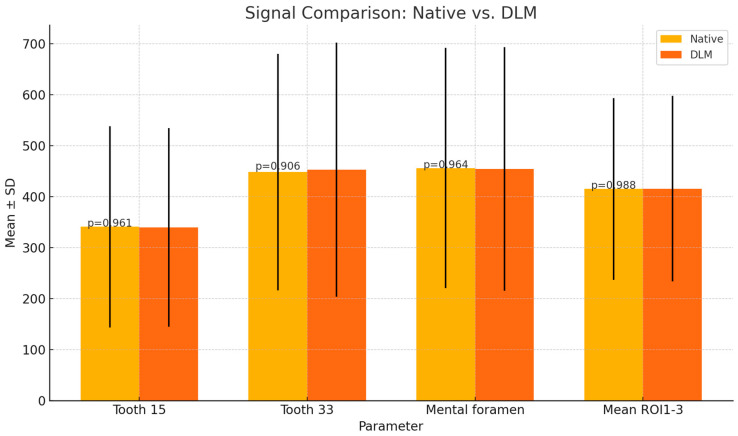
Results of the mean signal calculations (mean values error bars represent SDs). No statistically significant differences were found (*p* > 0.05).

The average signal measured in the regions of interest (ROIs) in three locations (periapical area of teeth 15 and 33 and spongious bone of the mandible in the area of the mental foramen) showed slightly lower mean values in DLM images than in native reconstructions. However, the difference was not statistically significant (*p* > 0.05). Table 1 summarizes the results of the objective image quality assessment. Graphical representation of the mean signal calculations in Figure 4.

**Figure 5:**
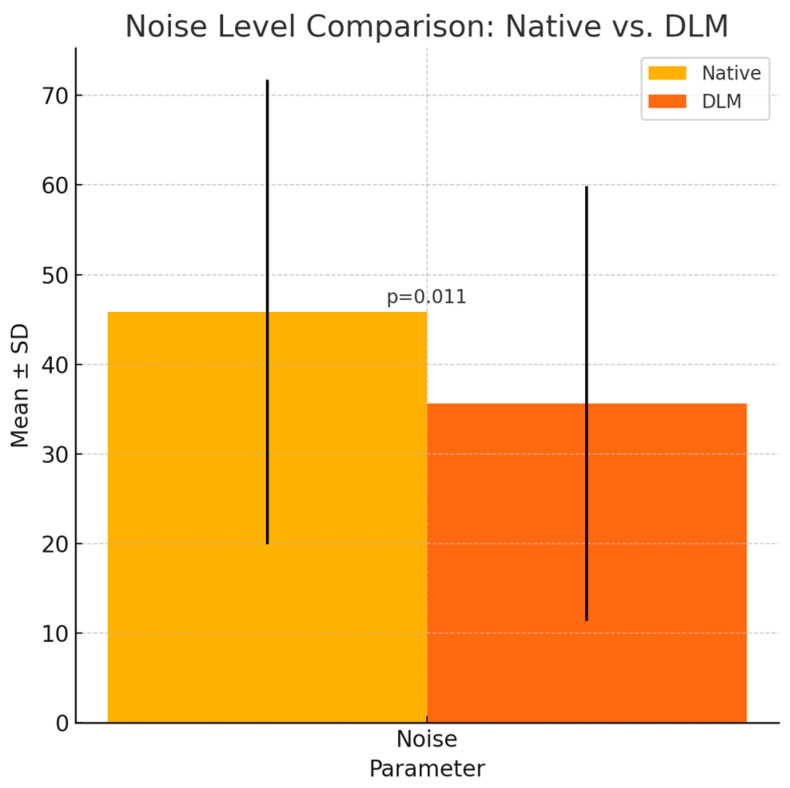
Results of noise calculations in ROIs 1-3 (mean values error bars represent SDs). *p* values shown on graphs. There was a statistically significant difference (*p* = 0.011).

There was a statistically significant difference between noise levels on both types of reconstructions (*p* = 0.011). Figure 5 illustrates mean noise levels.

**Figure 6:**
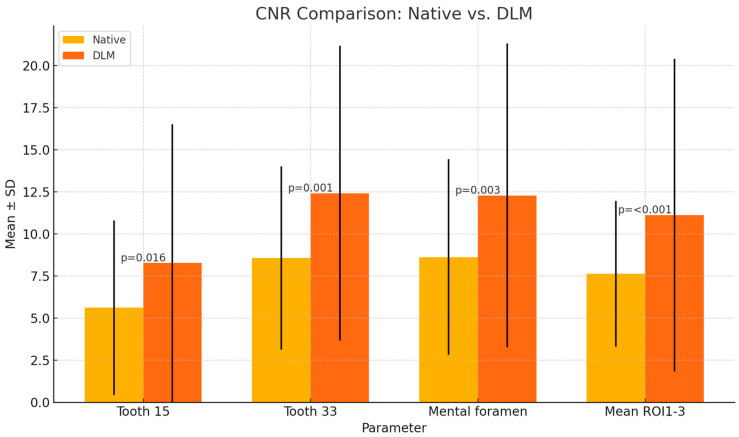
Results of CNR calculations (mean values, error bars represent SDs). *p* values shown on graphs.

The CNR in DLM reconstructions was significantly higher than that in native reconstructions across all examined locations (*p* < 0.05), as shown in Figure 6. The mean CNR in ROI_1-3_ in DLM images was 11.12 ± 9.29, while in the case of native reconstructions, it was 7.64 ± 4.33 (Table 1).

### 3.3. Subjective Image Quality

**Figure 7:**
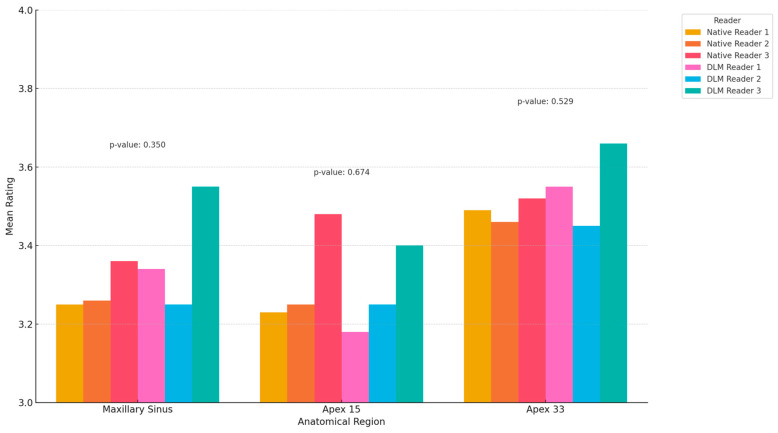
Results of subjective image quality assessments (mean values).

The results of the subjective image quality assessments are summarized in Table 2. The data in the table represent the mean ratings of all readers. Overall, subjective image quality was lowest for the apical area of tooth 15 in both the native and DLM reconstructions. The highest mean scores were given to the apical area of tooth 33 in both the evaluated reconstructions. The differences between the mean ratings for both types of reconstructions were slight and not statistically significant (*p* > 0.05), ICC = 0.753. Figure 7 presents the results of the subjective image quality assessments.

Inter-reader agreement for subjective image quality assessments was evaluated using Fleiss’ Kappa. The results indicated moderate to substantial agreement among the three readers, with Kappa values ranging from 0.536 to 0.628 for native reconstructions and 0.540 to 0.628 for DLM reconstructions (Table 3).

### 3.4. Error Study

Analysis of the repeatability of subjective image quality analysis carried out by the reader demonstrated excellent concordance (ICC = 0.841).

### 3.5. Sample Size

A power analysis was conducted to determine the appropriate sample size required to detect significant differences in noise levels between native and DLM reconstructions. Pooled SD of mean CNR values of DLM and native images was 7.25. The calculated Cohen’s d was 0.48.

The analysis indicated that a sample size of 34 subjects per group was sufficient to achieve a power of 0.8, with an effect size (Cohen’s d) of 0.48 and a significance level of 0.05. This sample size ensures that the study is adequately powered to detect meaningful differences in objective image quality parameters.

## 4. Discussion

The aim of this study was to assess the image quality parameters of standard dental CBCT images and images reconstructed using DLM algorithms. Our study revealed that DLM reconstructions had slightly greater mean signal values than native reconstructions, although this difference was not statistically significant. However, the CNR was significantly higher in the DLM reconstructions than in the native reconstructions. Noise levels were also statistically significantly lower in the DLM reconstructions than in the native reconstructions. This indicates that the evaluated DLM algorithm improves the contrast between the anatomical structures in CBCT images. The results of the subjective image quality analysis performed by three readers blinded to the type of reconstruction showed no statistically significant differences.

Surprisingly, although the differences were statistically insignificant, the results of the subjective image quality assessments showed mixed results in the evaluation of the selected anatomical structures. The mean scores for all readers of the alveolar recess of the maxillary sinus and the apical area of tooth 33 were both greater for DLM reconstructions than for native reconstructions. However, the ratings for the apical area of tooth 15 were greater in native reconstructions than in DLM reconstructions. In our opinion, this indicates a clear convergence in the quality of both reconstructions and high repeatability of readers in quality assessment. Upon re-evaluation by the readers, after the results of the analyses were obtained, some of the DLM reconstructions showed poorer delineation of structures, which might have influenced the image quality ratings of both periapical areas. However, combined with reduced noise levels, excessive smoothing of the very thin structures worsened the delineation of structures, for example, the periodontal ligament. This phenomenon might have an impact on the visualization of critical CBCT-indicated structures, such as the root canal or alveolar bone, where spatial resolution is key [30,31,32]. Similar results were shown by Ylisiurua et al. [33], who reported that deep learning algorithms enhanced the visualization of soft tissues but degraded the visualization of bones and teeth. The authors subjectively noted a significant decrease in resolution and concluded that the images resembled images reconstructed with “soft-tissue kernels” used with CT scanners. Since CBCT is used mainly in the diagnosis of bones and teeth, such over-smoothing of the details may compromise diagnostic accuracy. Future studies focused on evaluating the delineation of such structures may answer the question of whether DLM algorithms significantly reduce the value of the examination in assessing submillimeter structures.

Our findings suggest that although the quantitative improvements are noticeable, the qualitative assessment of these changes may require a higher threshold to achieve significance. We must emphasize that the evaluated DLM was not designed for CBCT imaging. The purpose of the program was to reduce additional image noise in CT images. Therefore, the results of our study should be regarded as a scientifically driven attempt to explore the impact of this tool on a domain similar to CT. The results are similar to our previous study evaluating the effects of applying the same vendor-agnostic DLM to CBCT images of TMJs [34]. The study showed significantly better objective image quality of DLM reconstructions compared to native images (CNR levels; *p* < 0.001). However, the results of subjective image analysis showed no significant differences in image quality between the reconstruction types (*p* = 0.055). Moreover, the assessment of degenerative TMJ lesions was not affected by the type of reconstructions assessed (*p* > 0.05). We concluded that the analyzed DLM reconstruction notably enhanced the objective image quality in TMJ CBCT images, but did not significantly affect the subjective image quality or DJD lesion diagnosis. Our studies provide new insights into the efficacy of the selected DLM in this specific context, separate from its general approval and usage. Therefore, we caution against the generalization of our results beyond this specific context. However, our findings indicate that the use of AI denoising algorithms designed for CT imaging may improve the objective image quality parameters of CBCT images. Further studies, including a larger number of examinations performed using various devices and different diagnostic protocols, could demonstrate greater differences in the results of qualitative and quantitative image assessments. It is likely that the results would be similar to those published on qualitative analyses of studies performed using low-dose protocols in standard CT examinations [22,31,35,36,37,38,39]. Compared with standard and iterative reconstructions (IRs), deep learning reconstructions have already proven to have the potential for radiation dose reductions between 30% and 71% while maintaining diagnostic image quality owing to improved noise reduction [40]. Nevertheless, the trend toward improved image quality with the use of DLM algorithms in CBCT is promising.

Recent studies [41,42,43] have assessed the effectiveness of generative AI in reducing noise and metal artifacts in dental CT images. Hegazy et al. (2020) [41] evaluated the image quality of low-dose dental CT images reconstructed with a generative adversarial network using the Wasserstein loss function (WGAN). The authors achieved both quantitative and qualitative improvements in image quality; however, interestingly, they encountered the problem of over-smoothing small image details. In a 2021 study [43], Hegazy et al. evaluated the impact of variations in the WGAN and U-WGAN on the image quality of half-scan dental CTs. Both the noise levels and qualitative image parameters were significantly improved in the AI-reconstructed images. Another notable study by Hu et al. [42] proposed a WGAN to decrease the level of noise and metal artifacts in low-dose dental CT images. The results of the study showed that the proposed WGAN algorithm effectively removed artifacts and noise from low-dose dental CT images and outperformed other methods, such as general GANs and convolutional neural networks, in terms of image quality and artifact correction.

The literature concerning noise optimization in dental CBCT examinations, as opposed to conventional CT, is limited. In a recent study by Ramage (2023) [18], the authors assessed the effect of standard filtered back projection (FBP) and iterative reconstruction (IR) on CBCT image noise. They found that compared with FBP, IR significantly reduced image noise (99.84 ± 16.28 and 198.65 ± 55.58, respectively). The authors concluded that the additional processing time for IR reconstruction was clinically acceptable. A study by Choi et al. [44] investigated the efficacy of a novel, self-supervised convolutional neural network in projection noise reduction. The phantom study revealed that the peak signal-to-noise ratio (PSNR) and structural similarity index measure (SSIM) significantly improved compared to those of uncorrected images—27.08 and 0.839 vs. 15.68 and 0.103, respectively. A similar phantom study by Han and Yu evaluated the efficacy of a novel self-supervising denoising method based on Bernoulli sampling [45]. The results showed that the proposed method outperforms conventional denoising methods by at least 4.47 dB in PSNR. Brendlin et al. [46] investigated the efficacy of deep-learning denoising (DLD) techniques in mitigating the trade-offs related to the radiation dose and noise of CBCT during interventional procedures. The results showed that the application of DLD enabled significant radiation dose reduction combined with enhanced objective image quality parameters (higher CNR and lower noise). Two studies evaluated the effectiveness of DLD techniques in maxillofacial CBCT [33,47]. Kim et al. confirmed that the use of DLD techniques improved the diagnostic accuracy of readers in diagnosing sinus fungal balls and chronic rhinosinusitis [47]. Ylisiura et al. [33] compared iterative and DLD techniques for effective noise reduction in dentomaxillofacial applications. Their study demonstrated that the proposed method enabled image enhancement comparable to that of the iterative method, but with faster processing time. However, despite promising results, the readers preferred iterative reconstruction over DLD images in hard tissue evaluation. However, none of these studies evaluated commercially available noise reduction methods, and the evaluated techniques were available only to narrow groups of scientists. Therefore, the possibility of comparing different DLD techniques is an exciting topic for further research.

The findings of this study suggest that the application of a DLM to dental CBCT images can improve the CNR without compromising diagnostic quality. These findings are supported by the objective measurements of the CNR, which showed a statistically significant improvement in the DLM-reconstructed images compared with the native reconstructions. It is important to note that while confidence intervals provide an estimate of the range within which the true parameter lies, they do not preclude the possibility of statistically significant differences between groups. Our findings of significant differences in CNR, despite overlapping confidence intervals, underscore the importance of hypothesis testing in statistical analysis. Compared to commercial software such as TrueFidelity™ by GE Healthcare and AiCE by Canon Medical Systems, our vendor-agnostic DLM offers several advantages. Unlike vendor-specific solutions, the vendor-agnostic DLM can be applied to scans from various manufacturers, enhancing its versatility in clinical settings. Although some studies have revealed that while the noise reduction capabilities of our DLM are comparable to those of commercial software, it excels in maintaining image quality across different imaging systems [48]. This flexibility could streamline workflows and reduce the costs associated with acquiring multiple software licenses.

However, this study has several limitations. The sample size, although adequate for a pilot study, was relatively small. Larger studies with more diverse patient populations are needed to generalize these findings. Moreover, the subjective nature of image quality assessment, even for experienced readers, can be influenced by individual biases. Although the study used predefined scales and illustrations to aid in the assessments, these evaluations are inherently subjective and should be interpreted with caution. Notably, this study focused on a specific DLM algorithm and CBCT scanner. Further research is required to evaluate the generalizability of these findings to other DLM algorithms and CBCT scanners. Additionally, we evaluated images acquired only with a “regular quality” preset; therefore, our findings cannot be extrapolated to other protocols, especially low-dose protocols.

## 5. Conclusions

Overall, the results of this study support the potential of DLMs to objectively improve CBCT image quality by increasing CNR and reducing image noise. However, some issues with the delineation of small bony structures were noted, although no statistically significant differences in subjective image quality ratings were found. Our results could have significant implications for patient care by reducing the radiation dose required for diagnostic-quality images and potentially improving the diagnostic accuracy of dentomaxillofacial pathology. Further research is warranted to fully understand the clinical impact of DLMs on CBCT and to explore their integration into standard practice.

## Figures and Tables

**Table 1:** Results of the objective image quality assessment.

Parameter		Native	DLM	*p*
Signal	Tooth 15	341 ± 197.60	339.91 ± 194.93	*p* = 0.961
	Tooth 33	448.33 ± 232.01	452.84 ± 249.1	*p* = 0.906
	Mental foramen	456.15 ± 235.78	454.46 ± 238.97	*p* = 0.964
	Mean ROI_1-3_	415.3 ± 178.11	415.74 ± 181.75	*p* = 0.988
Noise		45.83 ± 25.89	35.61 ± 24.28	*p* = 0.011 *
CNR	Tooth 15	5.62 ± 5.19	8.28 ± 8.25	*p* = 0.016 *
	Tooth 33	8.58 ± 5.45	12.42 ± 8.76	*p* = 0.001 *
	Mental foramen	8.63 ± 5.81	12.29 ± 9.02	*p* = 0.003 *
	Mean ROI_1-3_	7.64 ± 4.33	11.12 ± 9.29	*p* < 0.001 *

The signal and CNR are given as the means ± standard deviations. DLM—deep learning model reconstruction; ROI—region of interest; CNR—contrast-to-noise ratio. *—statistically significant difference.

**Table 2:** Results of the subjective image quality assessment.

Region	Native			DLM			*p*
	Reader 1	Reader 2	Reader 3	Reader 1	Reader 2	Reader 3	
Maxillary Sinus	3.25	3.26	3.36	3.34	3.25	3.55	0.350
Apex 15	3.23	3.25	3.48	3.18	3.25	3.40	0.674
Apex 33	3.49	3.46	3.52	3.55	3.45	3.66	0.529

DLM—deep learning model. *p*—Wilcoxon paired test.

**Table 3:** Inter-reader agreement for subjective image quality assessment.

Region	Reconstruction	ICC	Interpretation
Alveolar recess of maxillary sinus	Native	0.536	Moderate Agreement
	DLM	0.552	Moderate Agreement
Apex 15	Native	0.628	Substantial Agreement
	DLM	0.628	Moderate Agreement
Apex 33	Native	0.541	Moderate Agreement
	DLM	0.540	Moderate Agreement

DLM—deep learning model; ICC—interclass correlation coefficient.

## Data Availability

Data are available upon request.
